# The Impact of an Authoritarian Personality on Pro-Environmental Behaviour for Air Pollution Mitigation through Interactions with Social Norms

**DOI:** 10.3390/ijerph18179301

**Published:** 2021-09-03

**Authors:** Jiawen Cao, Jin Chen

**Affiliations:** 1 CAS Key Laboratory of Tropical Forest Ecology, Xishuangbanna Tropical Botanical Garden, Chinese Academy of Sciences, Mengla 666303, China; caojiawen@xtbg.ac.cn; 2 College of Life Science, University of Chinese Academy of Sciences, Beijing 100049, China

**Keywords:** air pollution, authoritarian, personality, pro-environmental behaviour, risk perception

## Abstract

This study examines how risk perception and authoritarian personality affect public engagement in mitigating air pollution. Data were collected (*n* = 2010) from 13 Chinese cities with varying air pollution gradients using questionnaires. The results demonstrated that air pollution was significantly correlated with people’s risk perception and concern about air pollution, which significantly affected their pro-environmental behaviour (PEB). However, high-risk perceptions undermine the public’s self-efficacy and reduce people’s PEB in the private sphere. People with high scores of the authoritarian personality type were reluctant to engage in PEB in the private sphere; interestingly, it can also be transformed into a stronger PEB in the public sphere via social norms. Thus, this study suggests that educational activities can break the negative link between authoritarianism and environmentalism, leading to behavioural change. Hence, it is essential for education programs to harvest positive outcomes via adaptive approaches for varying authoritarian personalities.

## 1. Introduction

Air pollution appears to be an inescapable phenomenon in the era of global industrialisation. Many developing countries have been suffering from air pollution for decades, including China [1]. Air pollution causes major health problems [2,3] and threatens a country’s economic sustainability [4,5].

Air pollution mitigation requires public support and engagement. However, individuals’ pro-environmental behaviour (PEB) is often a complex psychological decision-making process influenced by several factors. For example, studies have shown that public perception of the severity and risk of air pollution can lead to pollution mitigation behaviour [6,7,8,9]. Individual actions are also strongly influenced by personality [10,11]. For example, numerous studies have found that an authoritarian personality is related to a range of anti-environmental sentiments [12,13]. People who score higher in authoritarianism tend to be less convinced that climate change is happening or that humans contribute to the problem [14,15]; they tend to not believe that there are benefits from acting pro-environmentally [16]. Other studies have shown that authoritarians are more likely to agree that acting on environmental issues will be costly for their country [17]. Nevertheless, people with high scores in authoritarianism show a slight diversity in their approach to environmental issues; in general, they show negative attitudes toward the environment [18].

Therefore, more studies are required to understand how these psychological and demographic variables affect PEB formation, particularly in China, where air pollution problems are still severe, although great efforts have been made by the government [19], but studies on this topic are still lacking.

### 1.1. Dimension of PEB

PEB refers to behaviour that consciously seeks to minimise the negative impact of one’s actions on the natural and human-made world [20]. Early research on PEB presumed it to be a unitary and undifferentiated sum of behaviours, while more recent studies have suggested distinct types of environmentally significant behaviours. Moreover, different behaviours are determined by different combinations of causal factors [21,22]. Some behaviours are inherently more difficult to perform than others [23], and participation levels are influenced by various social and structural factors [24,25,26]. Most environmental and psychological studies have primarily highlighted PEB within the private sphere [27,28,29]. However, recent civic engagement from both a non-activist and activist standpoint in the public sphere has drawn attention to PEB research, such as environmental citizenship, policy support, and other environmental problems [30,31,32]. Therefore, to better understand their causal factors, PEB can be principally divided into PEB in the public sphere (Pu-PEB) and PEB in the private sphere (Pr-PEB) [33]. This two-dimensional division has been recognised and used by many scholars [31,34,35], and it was also adopted in this research.

### 1.2. Model Development

Building on Ajzen’s theory of planned behaviour, a classic theory for studying behavioural change, Fishbein [33] integrates individual as well as socio-cultural contextual factors (e.g., personality, intelligence, experience, age, gender, and culture) to determine behaviour by influencing behavioural attitudes, norms, and self-efficacy. Environmental factors and individual competence also moderate the occurrence of influential intentions to behaviour, which is in line with our research intentions. In our model, risk perception and authoritarian personality were taken as the external variables, and we tried to relate the external variables to behavioural change.

Many studies have shown that people perceive air pollution [36,37,38,39], and visibility and unpleasant odours often create the basis of public perceptions regarding air pollution [40]. Risk perception is one of the most important indicators of public concern over air pollution [41,42,43] and can be a determinant of PEB [44,45,46,47]. Witte [48,49] proposed that risk perception might consist of two distinct aspects: perceived susceptibility and perceived severity. Furthermore, severe air pollution has been reported to cause people to perceive a sense of powerlessness in abating the problem [50,51], thus undermining their self-efficacy. In this case, a high perceived air pollution risk may lead to a low PEB due to low self-efficacy [52].

The two questions addressed in this study were: (1) Can the public perceive the severity of air pollution in China? (2) To what extent can risk perception generate the public’s air pollution mitigation behaviour? Accordingly, we formulated the first two hypotheses:

**Hypothesis** **1** **(H1).** *The risk perception of air pollution is significantly correlated with the actual air pollution levels.*

**Hypothesis** **2** **(H2).** *High-risk perception of air pollution can generate public air pollution mitigation behaviour.*

Authoritarianism has traditionally been conceptualised as a tendency to submit to authority [53] and was first referred to as a superstitious, rigid, and conservative psychological personality [54]. Later, Altemeyer [53] subdivided authoritarian personality into three dimensions: obedience to authority, criticism of the disobedient, and adherence to traditional values, leading to the development of right-wing authoritarianism (RWA). Collectively, someone who scored high in authoritarianism will have (1) a greater need for order and, conversely, less tolerance for confusion or ambiguity, and (2) a propensity to rely on established authorities to provide that order. Stanley and Wilson [18] showed that authoritarianism has a considerable negative association with environmentalism, regardless of how authoritarianism is measured.

Social norms have been used to refer to common behaviours and beliefs that associate conformity to these behaviours, similarly to conformity performance [55,56,57]. A vast body of evidence demonstrates that social norms impact various behaviours, especially environmental behaviour (e.g., [57,58,59]). In particular, the Chinese may advocate collectivism more than the Western idea of individualism [60]. Under the long tradition of collectivist culture, Chinese households often follow significant people, including authorities and celebrities. From the definitions of authoritarian and social norms, the traits of high authoritarians are similar to the psychological roots of adherence to social norms; both groups fear confusion [61,62].

This section discusses the following questions: (1) Is authoritarian personality an effective predictor of PEB? (2) How does an authoritarian personality interact with a person’s social norms that could explain PEB? Therefore, we formulated the following hypotheses:

**Hypothesis** **3** **(H3).** *The authoritarian personality predicts low PEB.*

**Hypothesis** **4** **(H4).** *An authoritarian personality may interact with social norms, thus affecting people’s behaviour; authoritarian personalities with positive environmental and social norms can present high PEB.*

## 2. Materials and Methods

### 2.1. Research Sites and Participants

Referring to the literature on the spatial distributions and trends of the air quality index across China [63], we selected 13 cities with air quality gradients for this study. All sites are in a provincial capital city with a population of more than five million (see Table A1 of Appendix A for information of the study sites).

In this study, we conducted a questionnaire survey of parents of primary school students in the city. With the help of the Chinese Union of Botanical Garden, we chose one or two schools in each city. A total of 2731 parents from 21 primary schools participated in the survey. Regarding the consistency of positive and negative items and completeness, there were 2010 valid samples (*n* _male_ = 713, *n* _female_ = 1297). The effective sample rate was 73.5% (see Table A2 of Appendix A for detailed information about the samples).

For each city, data on actual air pollution were obtained from the China Bulletin on environmental quality issued by the Ministry of Environmental Protection, and the air quality in key environmental cities was obtained from the statistical yearbook published by China’s National Bureau of Statistics. Selected air quality composite indexes (the sum of the quotients of the concentrations of the six pollutants and the corresponding secondary standards during the evaluation period is the comprehensive ambient air quality index of the city for that period), and the concentration of major air pollutants as indicators of actual air pollution levels were collected for further analysis. The major air pollutants include PM_2_._5_, PM_10_, and SO_2_. The Ministry of Environmental Protection used the air quality composite index to rank the city’s ambient air quality. We collected air quality composite index data for the experimental cities for two years, from 2016 to 2017. We used the average of these two years to represent the actual air pollution levels of the experimental cities. Similarly, we used three years of average data of major air pollutant concentrations in the experimental cities from 2016 to 2018 (see Table A3 of Appendix A for specific data).

### 2.2. Measures of Key Constructs

The questionnaire is dependent on previous validation scales from the literature and has been localised and pretested. The survey was initially translated into Chinese (Mandarin) by the first author, inviting five professional EE educators to revise with an instruction to keep the original wording and meaning of the items as much as possible, meanwhile considering Chinese cultural background and expression habits. Prior to being administered in China, we carried out an interview with parents to see whether they could understand the meaning of each item. It is also important to note that this survey was approved by the Ethics Committee of the Ethics Committee of Xishuangbanna Tropical Botanical Garden (see Table A4, Table A5 and Table A6 of Appendix A for detailed information about the questionnaire). We conducted two pilot studies between April and May 2019 to improve the accuracy and conciseness of the questions. The first pilot study tested the parents of one primary school and one middle school in Chengdu, Sichuan Province (*n*_1_ = 109); the second test (*n*_2_ = 248) was conducted at a middle school in Kunming, Yunnan Province. The first pilot study found that the child-rearing scale had poor internal consistency reliability (Cronbach’s α = 0.403). Using exploratory factor analysis, we examined Pu-PEB and Pr-PEB, and the questionnaire was accepted with a Kaiser–Meyer Olkin statistic of 0.787 (*p* < 0.001). However, there were three principal components of PEB that emerged in 13 items. We deleted one component that included only two items and added another item. Subsequently, we were left with 11 PEB items. After conducting the second pilot, the reliability and validity of the questionnaire were deemed acceptable, except for the child-rearing scale.

#### 2.2.1. Risk Perception

Risk perceptions were measured based on the product terms of perceived susceptibility and the perceived severity of these threats [64]. Perceived susceptibility was assessed on a 5-point Likert scale ranging from 1 (strongly disagree) to 5 (strongly agree). Additionally, perceived severity was assessed on a 5-point Likert scale ranging from 1 (strongly disagree) to 5 (strongly agree). The six terms achieved good reliability (Cronbach’s α = 0.906).

#### 2.2.2. Authoritarian Personality

When scholars refer to the concept of authoritarianism, a measurement with high validation and reliability presents a challenge [65]. Recent studies have tried to overcome measurement problems by using a scale that gauges child-rearing preferences [66]. Specifically, researchers required respondents to judge attractive attributes in children, introducing the topic in the following way:

“Although there are several qualities that people feel that children should have, every person thinks that some are more important than others. I am going to read the pairs of desirable qualities. Please tell me which one you think is more important for a child to have. The pairs of attributes are independence versus respect for elders, obedience versus self-reliance, curiosity versus good manners, and being considerate versus well-behaved. Those who value ‘respect for elders,’ ‘obedience’, ‘good manners,’ and ‘being well behaved’ score highest of the scale, while those who value ‘independence’, ‘self-reliance,’ ‘curiosity’, and ‘being considerate’ score lowest.”

These indicators draw on a metaphor equating hierarchical thinking at home with hierarchical thinking in society: a person who prefers enforcing conformity in a child ought to favour enforcing conformity in social subordinates. Thus, in this study, we adopted a child-rearing scale to measure authoritarian personality. Two pilot studies found that this scale had a very poor internal consistency reliability. In our formal experiment, 4-term child-rearing had poor reliability (α = 0.169).

#### 2.2.3. Pro-Environmental Behaviours (PEB)

To measure Pu-PEB and Pr-PEB, we derived the items from Lu et al. [31]. We revised these items by referencing the 2003 Chinese General Social Survey on the environmentally friendly behaviour of urban residents and to incorporate real-life actions that might mitigate air pollution. The pilot questionnaire was designed based on the dimensions of Pr-PEB and Pu-PEB. Exploratory and confirmatory factor analyses were conducted to derive formal PEB constructs. The questionnaire for air pollution PEB in China consisted of 11 questions: 5 assessed Pr-PEB (Cronbach’s α = 0.673) concerning low-carbon travel, energy-saving, and reduced use of disposable items (e.g., ‘For short distances (up to 15 min), I choose to walk or go by bike’); Pu-PEB included six items (Cronbach’s α = 0.836; e.g., ‘Making complaints to local environmental authorities about air pollution issues around us, such as garbage incineration in the last year’). Participants were asked to indicate how often they engaged in each of the 11 specific behaviours in the previous year using 5-point scales ranging from 1 (never) to 5 (always).

#### 2.2.4. Proximal Variables Affecting Behaviour Change

We measured the proximal variables’ norms and self-efficacy that influenced the occurrence of PEB. Considering that public concern about air pollution is an important proximal variable influencing the public to generate mitigation behaviour, we incorporated concerns into the model. Concern measured seven terms used by Hu and Chen [17]; we deleted one item due to missing data for one city and attained good reliability (Cronbach’s α = 0.751).

In the original model of the theory of planned behaviour, social norms were mentioned as subjective norms. However, several researchers, such as Moan and Rise [67] and Cristea et al. [68], suggested incorporating descriptive norms with subjective norms to describe social norms. Descriptive social norms describe how most people behave (e.g., ‘my colleagues promote a low-carbon lifestyle’), while subjective norms refer to the perceived pressure from significant people to perform or refrain from behaviours [69] (e.g., ‘My family supports my complaint about behaviours that pollute the air—such as burning garbage’). The 6-term social norm, adapted from Shi et al. [60], attained good reliability (Cronbach’s α = 0.792) after pilot advising.

Self-efficacy refers to an individual’s belief in their performance or ability to perform tasks in a particular domain [70]. The seven items adapted from Lemée et al. [71] measured self-efficacy and achieved good reliability (Cronbach’s α = 0.617).

Personal norms represent one’s feelings of moral obligation towards acting [72]. As a measure of personal norms, the two constructs may shape air pollution mitigation actions. For example, those who regard pollution mitigation actions as morally right and those who anticipate feelings of guilt if they do not perform such actions have higher mitigation actions. Personal norm items followed these two aspects and references [72]; for example, ‘I would feel guilty if I did not engage in low-carbon travel in daily life’. After the pilot, ‘I feel morally obligated to bring a reusable water glass to work’ was changed to ‘I feel obliged to do my part to ensure my city has excellent air quality’. Six items had good reliability (Cronbach’s α = 0.767) and were more consistent with air pollution-related mitigation behaviour.

These four variables were rated on a 5-point Likert scale ranging from 5 (strongly agree) to 1 (strongly disagree).

#### 2.2.5. Demographic

The sociodemographic variables of gender, age, family monthly income (CNY), education, and environmental protection workers (yes/no) were included in our questionnaire [73].

### 2.3. Data Analysis

To compare urban dwellers’ risk perception of air pollution with actual air pollution, the data measuring actual air pollution, such as the air quality composite index and concentration of major air pollutants, were analysed using linear regression with public risk perception of air pollution. Additionally, our dataset included individual-level information nested within the city-level data. Therefore, we use the hierarchical linear model [74], which can analyse nested data, to investigate the influence of city-level and individual-level variables on public perception of air pollution. A scatter plot of the actual air pollution concentration indicators and perceptions was also created by Rstudio (v 1.2.5001, Boston, MA, USA) to visualise the relationship between objective air pollution levels and subjective public perceptions. Rstudio is an open source & professional software for data analysis (https://www.rstudio.com/, accessed on 28 March 2019).

To understand how changes in different PEB were related to the changes in risk perception and authoritarian personality, controlling the site as a random factor, multiple regression using a mixed linear model in Rstudio (v 1.2.5001) was employed. Three regression models were used: the sociodemographic, risk perception, and authoritarian personality variables were included in Model 1; changes in perceptual variables were included in Model 2; and sociodemographic, risk perception, and the authoritarian personality and perceptual variables were included in Model 3. Furthermore, a path model analysis was constructed using IBM SPSS Amos (*n* = 2010) to understand how risk perception and authoritarian personality indirectly influence Pr-PEB and Pu-PEB. Sociodemographic variables were included in the analysis as covariates.

The public’s risk perception of air pollution was classified into three levels according to their air pollution risk perception score: low-risk perception (City: Kunming, Xiamen, Fuzhou, Shenzhen, Nanning; *n* = 805), moderate-risk perception (City: Nanjing, Changsha, Chengdu, Zhengzhou; *n* = 566), and high-risk perception (City: Shijiazhuang, Taiyuan, Wuhan, Xian; *n* = 605). Univariate statistical models and pairwise comparisons with the Tukey HSD test were used to evaluate the differences between Pu-PEB and Pr-PEB in the differential risk perception region. Type III sum of squares was used to determine statistical significance (*p* < 0.05). Meanwhile, we used a multi-cluster analysis approach to compare the differences in path model analysis across risk perception regions.

## 3. Results

### 3.1. Risk Perception of Air Pollution

**Figure 1:**
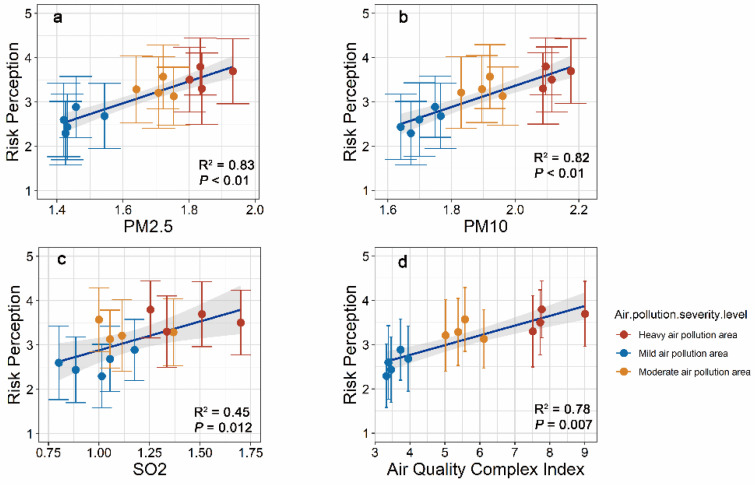
The relationship between PM_2_._5_, PM_10_, SO_2_ average concentration, air quality composite index, and air pollution risk perception. Note: Each spot represents one city, *n* = 13; the colour represents the degree of air pollution: blue—mild pollution, orange—moderate pollution, red—heavy pollution, and the error bar represents standard deviation. The abscissa is actual air pollution levels for the city: (**a**) Logarithm of the annual average concentration of PM_2_._5_ (μg/m^3^) from 2016 to 2018, (**b**) Logarithm of the annual average concentration of PM_10_ (μg/m^3^) from 2016 to 2018, (**c**) Logarithm of the annual average concentration of SO_2_ (μg/m^3^) from 2016 to 2018, (**d**) 2017–2018 Average air quality composite index (evaluation period, six pollutants concentration, and the sum of the corresponding secondary standard of business is the city’s air quality composite index of the period, the ecological environmental protection for urban ambient air quality ranking), and the ordinate is the risk perception of subjective air pollution.

Urban dwellers’ risk perception of air pollution is generally consistent with actual air pollution data (Table 1), supporting H1. A multi-layer linear model confirmed that under the control of demographic variables, all the actual air pollution measurement indicators (PM_2_._5_, PM_10_, SO_2_, and air quality composite index) were significantly positively correlated with people’s risk perception of air pollution. We also tested the relationship between risk perception and actual air pollution in different cities. All cities demonstrated similar results, showing that air quality indicators had a significant positive correlation with perceived air pollution (Figure 1).

### 3.2. The Relationship between Risk Perception, Authoritarian Personality, and Pro-Environmental Behaviour

The mixed linear model analysis (Table 2 and Table 3) showed that different causal variables work differently to influence the two PEBs. For Pu-PEB, concern and social norms positively predicted Pu-PEB. However, personal norms and self-efficacy failed to explain Pu-PEB. Risk perception and authoritarian personality did not show a significant direct relationship with Pu-PEB, indicating that H2 and H3 were not supported (Table 2). Demographic variables (gender, age, and education) were significant predictors of Pu-PEB. Furthermore, men reported stronger Pu-PEB than women, and younger and more educated people showed higher Pu-PEB. For Pr-PEB, risk perception and authoritarian personality were negatively correlated with Pr-PEB in Model 3 (Table 3), indicating that H2 and H3 were supported. Self-efficacy, personal norms, and concerns were also significant predictors of Pr-PEB. Among the demographic variables tested, gender and education were significant predictors of Pr-PEB, women reported stronger Pr-PEB than men, and higher education was associated with higher Pr-PEB.

**Figure 2:**
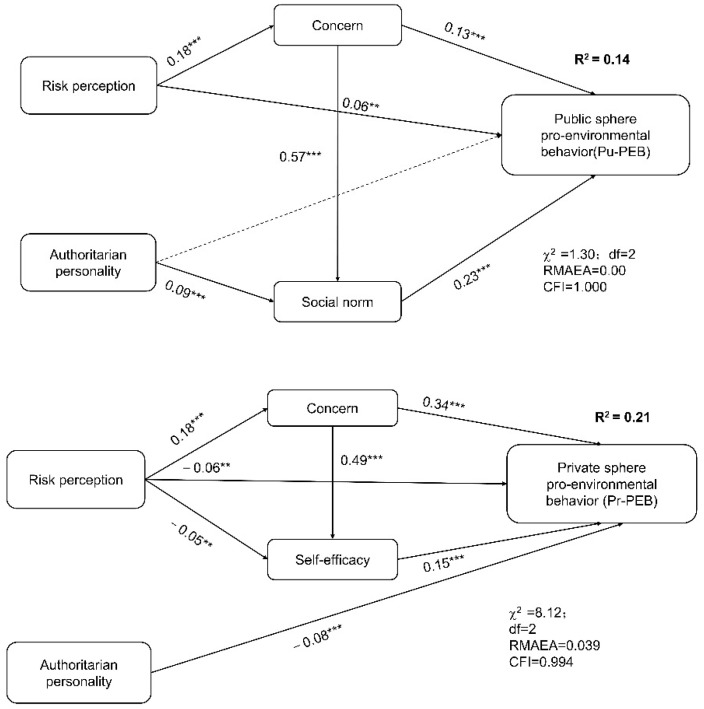
The relationship between risk perception, authoritarian personality, and pro-environmental behaviour both in private and public spheres. Note: The solid line represents significant influence, while the dashed line represents no significant influence. ** *p* < 0.01, *** *p* < 0.001.

The path analysis models (Figure 2) showed that risk perception could trigger public concern about air pollution, significantly affecting Pr-PEB and Pu-PEB. Risk perception could directly predict Pu-PEB but directly negatively predict Pr-PEB. Risk perception could also be mediated by self-efficacy in Pr-PEB; high risk leads to low self-efficiency, thus causing a low Pr-PEB.

Authoritarian personality has a significant negative effect on Pr-PEB. However, for Pu-PEB, authoritarian personality indirectly positively impacts Pu-PEB via social norms; authoritarian personalities have high social norms and high Pu-PEB (Figure 2).

### 3.3. PEB in Different Risk Perception Areas

**Figure 3:**
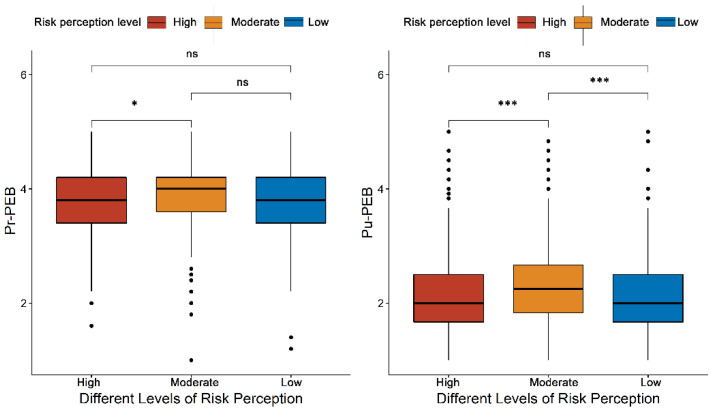
The difference between pro-environmental behaviours in private and public spheres in regions with different levels of air pollution risk perception: ns represents no significant difference. Note: * *p* < 0.05, *** *p* < 0.001.

Our data showed that risk perception is an important factor influencing people’s air pollution mitigation behaviour. Therefore, we investigated whether there was a difference in people’s air pollution mitigation behaviour in different risk perception areas. Multiple comparison procedure results revealed that Pu-PEB was significantly higher in moderate risk perception areas than in low- and high-risk perception areas (Figure 3). For the Pr-PEB, the highest value was also in the area with moderate risk; however, the difference between the moderate- and high-risk perception areas was not significant (Figure 3).

We found that the air pollution risk perception variable (β = 0.117, *p* < 0.05) significantly influenced Pu-PEB directly through the different risk perception region group analysis only under the condition of moderate risk perception. In the high- and low-risk perception areas, risk perception of air pollution did not influence Pu-PEB (β = 0.016, *p* > 0.05; β = 0.033, *p* > 0.05) (Table 4).

## 4. Discussion

With 2010 valid samples from 13 Chinese cities, this study presents interesting patterns on people’s perception of air pollution and pollution risk and the psychological variables affecting their Pu-PEB and Pr-PEB. As predicted, the degree of air pollution can be indicated by people’s perceptions. Authoritarian personality is also a strong predictor of PEB in complex and contradictory ways. High scores on authoritarian measures predicted a low Pr-PEB but could lead to a high Pu-PEB via the mediation of social norms. Surprisingly, the highest PEB for both the private and public spheres occurred in cities with moderate pollution perceived risk.

A significant congruence between actual air pollution and air pollution risk perception was presented in this study, which supported our hypothesis. This result is similar to those of previous studies conducted in China [39]. However, they contrast with the findings of studies from many developed countries that have identified an actual–perceived mismatch in air pollution [37,75]. This inconsistency might be because air pollution is highly tangible and visible and often affects the human senses in many Chinese cities, and air pollution problems are much more severe than those in the study areas of developed countries. For example, Kim, Yi, and Kim [37] found that the average PM_2_._5_ concentration was 57.86 ± 7.62 μg/m^3^. However, our data showed that the highest PM_2_._5_ concentration in high perception areas was 85.67 μg/m^3^.

Additionally, air quality data have been incorporated into weather forecasts on mobile phones in many cities in China, enhancing the visibility of air quality to the public. Moreover, wide-ranging discussions on air pollution problems by the Chinese social media are being initiated, and people are becoming increasingly aware of pollution issues in China.

In this study, we determined that Pr-PEB and Pu-PEB are influenced by different causal variables. The Pu-PEB was directly influenced by concern and social norms, while Pr-PEB was directly influenced by concern, personal norms, self-efficacy, authoritarian personality, and risk perception. Concern is a significant predictor of both Pr-PEB and Pu-PEB. Concern about environmental problems has been repeatedly reported to strongly predict PEB occurrence [76,77,78,79]. Pr-PEB can be easily influenced by personal capabilities [22]; therefore, self-efficacy is a good predictor of such behaviour. Several studies have proven the influence of personal norms on PEB [80,81]. However, in our study, personal norms significantly influenced Pr-PEB but not Pu-PEB. This result is broadly consistent with the scepticism of many researchers regarding the normative activation model. Previous studies have found that personal norms can successfully explain low-cost environmental behaviour. However, its explanatory power appears insufficient when the cost of behaviour is relatively high, such as effectiveness, money, time, and other factors [82]. Social norms can significantly and positively predict Pu-PEB but have no predictive effect on Pr-PEB. In China, ecological civilisation has also been proposed as a national development strategy. Therefore, Pr-PEB, such as recycling plastic bags and choosing public transportation, may have been internalised into personal norms as they are continually reinforced by social norms or personal effort. Research has also shown that social norms can be a better predictor of PEB occurrence by further internalising personal norms [22,83,84]. Another reason for the social norm positively predicting Pu-PEB could be the tendency to meet social expectations. People tend to fit into society and seek social respect. They are also most likely to adopt behaviours that others find effective, thus performing corresponding PEB in the public sphere.

The perceived risk of pollution did not lead to a linear correlation with PEB; instead, the highest PEB occurred in cities with moderate risk perception. Previous research also suggested that there may be an optimal level of risk perception associated with behaviour change [85] and that our moderate risk perception areas could be where such optimal perceptions exist. If individuals perceive the threat level to be too high, they become overwhelmed; likewise, if the perceived threat level is too low, it will cause them to ignore the risk. Combined with the results of the multi-cluster analysis, our study shows that a moderate level of risk perception is the risk perception mostly associated with behaviour change.

This study showed that authoritarian personality could negatively predict Pr-PEB but positively and indirectly determine Pu-PEB via social norms. Many studies have indicated a negative relationship between authoritarianism and environmental attitudes [13,86]. However, no studies have demonstrated the relationship between authoritarian personality and PEB. Our study found that authoritarian personality can significantly and negatively influence Pr-PEB. Vail et al. [87] reported that authoritarians are less likely to assume responsibility for acting on environmental issues. This finding might be because people who score high on authoritarianism are low in Pr-PEB.

Interestingly, this study showed that an authoritarian personality could positively and indirectly determine Pu-PEB via social norms. Hetherington and Weiler [61] suggested that authoritarianism ultimately stems from people’s efforts to reduce cognitive load. For example, people with high scores on authoritarian personality measures have a greater need for order and a lower tolerance for chaos or ambiguity, leading them to defer authority. The degree to which social norms influence behaviour may depend on an individual’s level of cognitive attrition, and the public chooses to follow social norms to reduce cognitive load. Thus, highly authoritarian individuals are more susceptible to the influence of social norms. Social norms can break the negative correlation between authoritarianism and environmental attitudes.

Furthermore, empirical studies have shown that if the netizen group with a high score in authoritarianism considers both official and unofficial media highly reliable, they will choose to trust and accept both types of media uncritically [88], and these two kinds of media are similar to social norms. However, whether the pattern presented here could also occur outside China requires further investigation. China has a long history of being influenced by Confucianism. Therefore, respecting and obeying those with a high status (authority) is highly valued in Chinese society. Regarding explicit behaviours, the Chinese usually show respect to and obey authority. Previous literature has indicated that the Chinese have a high degree of authoritarian personality, which is considered a national character [89,90].

Several limitations of the current study should be addressed in future research. First, our sample size was small compared to the Chinese population. Second, in this study, the path analysis models only provided about 21% and 14% of explanations for PEB, which indicated that confounding variables that explain the changes in behaviour should be considered in future research. Third, this study used the child-rearing scale to measure authoritarianism, which has not been used in China before and has low scale reliability. This is an ongoing problem when designing the scale and hinders the investigation of the introduction of the child-rearing scale. In future research, the use of other scales to test authoritarian personalities is required. Fourth, our results highlight that authoritarianism may shape people’s pro-environmental behaviour through social norms. However, we also test the significance of the effect of authoritarianism via the path analysis models for different cities; only four cities (Nanjing, Nanning, Xi’an, Xiamen) are significant. This implies that we need to add more cities to support the results of future studies.

Given the current severe air pollution, the Chinese government and scientists actively respond to and implement mitigation measures to address this environmental problem, for instance, the Action Plan on Prevention and Control of Air Pollution Introducing Ten Measures by the State Council of China in 2013 and introduced the Blue Sky Defense in 2017 to improve air quality. In addition to the government’s efforts to develop a legal system using technological tools or top–down approaches, it will also be necessary to encourage more daily actions from residents to combat air pollution. Personality and other psychological variables, such as risk perception, are often remote variables that influence behaviour [34]. Therefore, it is crucial to establish the relationship between these distal influencing variables and behaviour changes. This study demonstrated the link between the public’s perception of the risk of air pollution and the role of personality traits in mitigation actions. This is essential for the intervention of educational programs to promote public concern and engagement with the environment.

## 5. Conclusions

The current study, albeit in the context of China and Chinese culture, has broader implications for environmental education activities. First, our work empirically confirmed a significant congruence between perceived air pollution and actual air quality. This result suggests that the air quality of different localities in China still plays a vital role in forming individual air pollution perception, thereby their mitigation behaviour. In the future, the Chinese government should be more active in promoting public awareness of air pollution issues through social media and mobile air pollution monitoring systems. A timely and accurate publication of air pollution information would encourage the public to have a correct risk perception of air pollution and stimulate positive environmental behaviour.

Second, our study provides insights for recognising different types of environmental behaviours determined by different combinations of causal factors. Pr-PEB is largely influenced by self-efficacy, whereas Pu-PEB is more influenced by social norms. Thus, we can improve Pr-PEB by enhancing self-efficacy and Pu-PEB by enhancing social norms, thus helping to promote PEB from both the public and private spheres in a more targeted approach. Therefore, for Pr-PEB, it is very important to provide more information about the contribution of personal behaviour to environmental problems and to stress that people can solve a large part of the environmental problems by themselves. More specifically, the government should implement countermeasures according to the air pollution situation in different areas. People in areas with serious air pollution can perceive the impact of pollution more intuitively. Therefore, in these areas, the government should emphasise the self-efficacy of the people by demonstrating the results of governance to the public and encouraging them to improve the air pollution situation through Pr-PEB. For people in areas with less serious air pollution, the government should focus on building people’s environmental awareness and social norms, and it should also pay attention to air pollution and lay emphasis on Pu-PEB through publicity and education.

Third, the results of this study establish associations between distal variables such as personality, risk perception, and pro-environmental behaviour. Authoritarianism is associated with PEB but not necessarily negatively related to PEB, and PEB could be enhanced, particularly in the public sphere, by improving the social norms of environmental protection. Thus, in collectivistic and socially cohesive societies such as China, the role of authoritarianism and the propensity to rely on established authorities to provide that order might be a predictor for PEB by social norms. In other words, our results demonstrate that social norms can break a strong negative link between authoritarian personality and environmental attitudes. Therefore, it is very important to pay more attention to social norms in future environmental activities.

## Figures and Tables

**Table 1:** The influence of demographic and actual air pollution variables on air pollution risk perception.

Variables	Model 1	Model 2	Model 3	Model 4
	PM2.5 Annual Average (log)	PM10 Annual Average (log)	SO2 Annual Average (log)	Air Quality Composite Index
	β (SE)	β (SE)	β (SE)	β (SE)
Fixed effects				
Gender (‘1′ Male, ‘2′ Female)	0.04 (0.03) *	0.04 (0.04)	0.04 (0.02) *	0.04 (0.02) *
Education	0.04 (0.03)	0.04 (0.02) *	0.04 (0.03)	0.04 (0.02)
Years	−0.05 (0.04)	−0.05 (0.03)	−0.05 (0.04)	−0.05 (0.04)
Income	0.01 (0.02)	0.01 (0.02)	0.01 (0.02)	0.06 (0.02)
Environmental protection professional (‘1′ Yes)	0.04 (0.03)	0.04 (0.04)	0.04 (0.03)	0.04 (0.03)
Actual air pollution	2.43 (0.37) ***	2.43 (0.43) ***	1.17 (0.03) **	0.21 (0.03) ***
GDP per capita (log)	−0.00 (0.00)	−0.00 (0.00)	−0.00 (0.00)	−0.00 (0.00)
Random effects				
Intercept	0.21 ***	0.23 ***	0.376 ***	0.245 ***
Observations	2010	2010	2010	2010
Number of groups	13	13	13	13

Note: * *p* < 0.05, ** *p* < 0.01, *** *p* < 0.001.

**Table 2:** Predicting changes in public sphere pro-environmental behaviour to air pollution by demographic variables and social–psychological variables.

	Public Sphere Pro-Environmental Behaviour (Pu-PEB)								
	Model 1			Model 2			Model 3		
*Predictors*	*Estimates*	*CI*	*p*	*Estimates*	*CI*	*p*	*Estimates*	*CI*	*p*
(Intercept)	0.58	0.48–0.68	**<0.001**	−0.29	−0.41–−0.17	**<0.001**	−0.32	−0.46–−0.17	**<0.001**
Gender (‘1′ Male, ‘2′ Female)	−0.04	−0.06–−0.01	**0.016**				−0.05	−0.08–−0.02	**<0.001**
Age	−0.03	−0.06–−0.00	**0.040**				−0.04	−0.06–−0.01	**0.006**
Education	0.03	0.01–0.04	**<0.001**				0.02	0.01–0.04	**<0.001**
Income	0.00	−0.01–0.02	0.880				0.00	−0.01–0.02	0.680
Child rearing	0.03	−0.03–0.10	0.264				0.00	−0.05–0.06	0.905
Risk perception	0.04	0.02–0.05	**<0.001**				0.01	−0.00–0.03	0.139
Concern				0.12	0.09–0.16	**<0.001**	0.12	0.09–0.15	**<0.001**
Personal norm				0.01	−0.02–0.05	0.453	0.02	−0.02–0.06	0.324
Social norm				0.15	0.11–0.18	**<0.001**	0.15	0.11–0.18	**<0.001**
Self-efficacy				−0.02	−0.05–0.02	0.346	−0.02	−0.05–0.02	0.330
Random Effects									
σ^2^	0.10			0.08			0.08		
τ_00 city_	0.00			0.00			0.00		
ICC	0.05			0.03			0.04		
N _city_	13			13			13		
Observations	2010			2010			2010		
Marginal R^2^/Conditional R^2^	0.022/0.068			0.146/0.167			0.160/0.190		

Note: Bolded means stasticaly significant, *p* < 0.05.

**Table 3:** Predicting changes in private sphere pro-environmental behaviour to air pollution by demographic variables and social–psychological variables.

	Private Sphere Pro-Environmental Behaviour (Pr-PEB)								
	Model 1			Model 2			Model 3		
*Predictors*	*Estimates*	*CI*	*p*	*Estimates*	*CI*	*p*	*Estimates*	*CI*	*p*
(Intercept)	3.55	3.36–3.73	**<0.001**	1.55	1.33–1.77	**<0.001**	1.49	1.24–1.74	**<0.001**
Gender (‘1′ Male, ‘2′ Female)	0.14	0.08–0.19	**<0.001**				0.10	0.06–0.15	**<0.001**
Age	0.04	−0.01–0.09	0.108				0.02	−0.02–0.07	0.360
Education	0.06	0.04–0.09	**<0.001**				0.05	0.03–0.07	**<0.001**
Income	−0.01	−0.04–0.02	0.445				−0.01	−0.03–0.02	0.482
Child rearing	−0.08	−0.19–0.03	0.175				−0.11	−0.20–−0.01	**0.037**
Risk perception	0.02	−0.01–0.05	0.247				−0.03	−0.06–−0.00	**0.029**
Concern				0.35	0.29–0.41	**<0.001**	0.34	0.28–0.39	**<0.001**
Personal norm				0.10	0.04–0.17	**0.002**	0.10	0.03–0.16	**0.003**
Social norm				0.03	−0.03–0.09	0.351	0.04	−0.02–0.10	0.177
Self-efficacy				0.13	0.06–0.19	**<0.001**	0.13	0.07–0.19	**<0.001**
Random Effects									
σ^2^	0.31			0.26			0.25		
τ_00 city_	0.01			0.01			0.01		
ICC	0.04			0.04			0.03		
N _city_	13			13			13		
Observations	2010			2010			2010		
Marginal R^2^/Conditional R^2^	0.034/0.068			0.195/0.227			0.222/0.246		

Note: Bolded means statically significant, *p* < 0.05.

**Table 4:** The non-standardised regression coefficient and significance of each risk perception area in the simultaneous analysis of several groups.

Dependent Variable		Independent Variable	High-Risk Perception Area	Moderate-Risk Perception Area	Low-Risk Perception Area
			B (SE)	B (SE)	B (SE)
Concern	←	Risk perception	0.228 (0.025) ***	0.128 (0.031) ***	−0.011 (0.022)
Social norm	←	Child rearing	0.240 (0.079) **	0.227 (0.089) *	0.169 (0.064) **
Social norm	←	Concern	0.545 (0.036) ***	0.543 (0.039) ***	0.502 (0.031) ***
Public sphere’s pro-environmental behaviour (Pu-PEB)	←	Social norm	0.266 (0.057) ***	0.453 (0.061) ***	0.305 (0.046) ***
Public sphere’s pro-environmental behaviour (Pu-PEB)	←	Concern	0.172 (0.063) **	0.233 (0.063) ***	0.256 (0.047) ***
Public sphere’s pro-environmental behaviour (Pu-PEB)	←	Child rearing	−0.134 (0.117)	0.075 (0.122)	−0.107 (0.085)
Public sphere’s pro-environmental behaviour (Pu-PEB)	←	Risk perception	0.016 (0.039)	0.117 (0.037) **	0.033 (0.026)

Note: * *p* < 0.05, ** *p* < 0.01, *** *p* < 0.00.

## Data Availability

The original questionnaire data presented in this study are available on request from the corresponding author. And the data presented in this study are available in notes of Appendix A material Table A1 and Table A3.

## Appendix A

**Table A1:** Information for the study sites.

City	Longitude	Latitude	Number of Permanent Residents (10,000) ^a^	Air Quality Composite Index ^b^
Xiamen, Fujian Province	118°12′ E	24°47′ N	100.7	3.33
Xiamen, Fujian Province	118°12′ E	24°48′ N	100.7	
Xiamen, Fuzhou Province	119°31′ E	26°05′ N	83.6	3.39
Shenzheng, Guangdong Province	113°94′ E	22°51′ N	149.16	3.47
Kunming, Yunnan Province	102°66′ E	25°07′ N	87.82	3.74
Kunming, Yunnan Province	102°78′ E	24°93′ N	91.39	
Nanning, Guangxi Province	108°35′ E	22°83′ N	76.70	3.95
Nanning, Guangxi Province	108°39′ E	22°81′ N	76.70	
Nanning, Guangxi Province	108°29′ E	22°87′ N	81.34	
Changsha, Hunan Province	113°18′ E	28°36′ N	108.9	5.02
Nanjing, Jiangsu Province	118°83′ E	32°03′ N	100.3	5.38
Wuhan, Hubei Province	114°30′ E	30°64′ N	96.27	5.58
Chengdu, Sichuan Province	104°22′ E	30°63′ N	91.42	6.12
Zhengzhou, Henan Province	113°58′ E	34°81′ N	29.76	7.52
Taiyuan, Shanxi Province	112°57′ E	37°92′ N	43.72	7.73
Taiyuan, Shanxi Province	112°59′ E	37°80′ N	82.9	
Xi’an, Shaanxi Province	109°01′ E	34°26′ N	64.13	7.77
Xi’an, Shaanxi Province	108°91′ E	34°23′ N	134.32	
Shijiazhuang, Hebei Province	114°51′ E	38°04′ N	84.79	9.01
Shijiazhuang, Hebei Province	114°50′ E	38°14′ N	70.33	
Shijiazhuang, Hebei Province	114°50′ E	37°99′ N	84.79	

^a^ Data from the 2018 City District Government Report. ^b^ The air quality composite index is a tool used by the Ministry of Environmental Protection of the People’s Republic of China to evaluate and rank the air quality of 74 key cities. Retrieved from http://www.mee.gov.cn/hjzl/ (accessed on 28 March 2019).

**Table A2:** Demographic information for the valid questionnaires (*n* = 2010).

Demographic Information	Frequency	Percentage	Demographic Information	Frequency	Percentage
Gender			Family monthly income (CNY)		
Male	713	35.5	<3000	220	10.9
Female	1297	64.5	3001–5000	445	22.1
Age			5001–10,000	670	33.3
20–39	1062	52.9	10,001–30,000	543	27.0
40–59	933	46.4	≥30,000	132	6.4
≥60	15	0.7	Owns a private car		
Period of Residence			Yes	1568	78.9
<1	2	0.0	No	397	20.0
1	21	1.0	Occupation		
2	17	0.8	Students	20	1
3	28	1.4	Civil servant	80	4
4	21	1.0	Staff of public institutions	313	15.7
5	46	2.3	Self-employed entrepreneurs	298	14.9
6	313	15.6	Farmer	90	4.5
7	41	2.0	Company employee	544	27.3
8	53	1.6	Materfamilias	146	7.3
9	27	1.3	Retirement	10	0.5
10	172	8.6	Unemployed	12	0.6
>10	1237	6.2	Worker	102	5.1
Education			Other	379	19.0
Primary education	361	18.0	Environmental protection worker		
Secondary education	475	23.6	Yes	386	19.4
Higher education	1174	58.4	No	1595	80.4

**Table A3:** Air quality in the experimental cities (2016–2018 three-year average).

Pollution Level Classification	City	Annual Average SO_2_ Concentration (μg/m^3^) ^1^	Annual Average NO_2_ Concentration (μg/m^3^) ^1^	Annual Average Concentration of Respirable Particulate Matter (PM_10_) (μg/m^3^) ^1^	95% Daily Mean CO Concentration (mg/m^3^) ^1^	90% Daily Maximum 8 h O_3_ Concentration (μg/m^3^) ^1^	Annual Average Concentration of Respirable Particulate Matter (PM_2_._5_) (μg/m^3^) ^1^	Number of Days with Air Quality at or Better than Level 2 (Day) ^1^	Air Quality Composite Index ^2^	GDP Per Capita (Yuan) ^3^
Heavy	Shijiazhuang (sjz)	32.33	54.00	149.67	3.37	192.00	85.67	158	9.01	60,439.05
Heavy	Zhengzhou (zz)	21.67	53.33	122.33	2.27	190.00	69.00	164	7.52	109,322
Heavy	Xian (xa)	18.00	55.67	124.67	2.70	175.67	68.33	186	7.77	83,670.63
Heavy	Taiyuan (ty)	50.33	50.67	130.33	2.57	172.00	63.33	193	7.73	91,429.63
Moderate	Chengdu (cd)	11.33	51.67	91.33	1.63	168.67	56.67	233	6.12	96,743.77
Moderate	Wuhan (wh)	10.00	47.67	83.33	1.63	158.33	52.67	247	5.58	157,231.4
Moderate	Nanjing (nj)	23.33	45.00	78.67	1.53	181.33	43.67	252	5.38	173,057.9
Moderate	Changsh (cs)	13.00	37.33	67.67	1.33	154.67	51.00	269	5.02	144,784
Mild	Nanning (nn)	11.33	34.00	58.33	1.33	120.33	35.00	342	3.95	52,098.46
Mild	Shenzhen (sz)	7.67	30.67	43.67	1.00	139.33	27.00	347	3.47	541,507.5
Mild	Fuzhou (fz)	6.33	28.33	50.00	0.97	136.00	26.33	349	3.39	101,807.8
Mild	Xiamen (xm)	10.33	31.33	47.00	0.87	115.33	26.67	361	3.33	188,817
Mild	Kunming (km)	15.00	31.33	56.00	1.30	125.33	28.67	361	3.74	84,891

^1^ Data on air pollution and the number of days with good air quality from the National Bureau of Statistics of China-City Statistical Yearbook: http://www.citypopulation.de (accessed on 27 March 2019). ^2^ The air quality composite index is a tool used by the Ministry of Environmental Protection of the People’s Republic of China to evaluate and rank the air quality of 74 key cities: http://www.mee.gov.cn/hjzl/ (accessed on 28 March 2019). ^3^ GDP per capita data from China’s National Bureau of Statistics: http://data.stats.gov.cn/ (accessed on 27 March 2019).

**Table A4:** Questionnaire A.

Questions A	
Measure	Questions
Air pollution perception	How severe is the air pollution in the city you live in?
	2. What is your perceived number of good air quality days in your city of residence in the past year?
Risk perceptions (susceptibility * severity) (α = 0.906)	I think the air pollution in my city has affected people’s health.
	2. I think the impact of air pollution in my city on people’s daily life is extremely minimal.
	3. The air conditions in our city are so bad that sometimes it makes it hard to breathe.
	4. I think the air quality in my city can have a negative impact on the health of children and pregnant women.
	5. The air quality in our cities can have a negative impact on people’s life expectancy.
	6. I think my health and my family’s health is vulnerable to the air pollution in our city.
Private-sphere PEB (Pr-PEB) (α = 0.678)	In order to mitigate air pollution, I choose public transportation instead of driving when conditions permit.
	2. For short distances (up to 15 min), I choose walk or go by bike.
	3. In order to reduce the use of disposable products in my daily life, I will bring my own water cup and shopping bags for travel or shopping.
	4. I choose not to use disposable chopsticks when I go out to eat.
	5. I will pay attention to saving energy in my life, such as turning off the lights when people leave.
Public-sphere PEB (Pu-PEB) (α = 0.836)	Sharing or commenting on information related to air pollution in my circle of friends.
	2. Following the announcement of environmental litigation and environmental policy consultation on the government website and attending related events.
	3. Making complaints to local environmental authorities about local issues of air pollution, such as garbage incineration.
	4. Supporting environmental organizations in their lawsuits or environmental projects against environmental pollution by donations or other means.
	5. Using social media to expose environmental pollution incidents.
	6. Taking the initiative to participate in local environmental volunteer activities.
Concern (α = 0.751)	I usually pay attention to the media or online reports and information about air pollution.
	2. Normally, I would discuss air pollution-related issues with people around me.
	3. I tried to understand how to reduce air pollution.
	4. To mitigate air pollution, I will care about the use of energy in my life (e.g., electricity use).
	5. I think I need to take some actions (such as taking public transportation more often) to slow down the air pollution.
	6. I rarely think about air pollution.
Personal norm (α = 0.767)	I feel a moral obligation to conserve energy (such as saving electricity) no matter what other people do.
	2. I would feel guilty if I did not use low-carbon transport in daily life.
	3. I feel a moral obligation to share what I see about air pollution in my circle of friends so that more people can learn about it.
	4. I feel a moral obligation to persuade people around me to pay attention to the problem of air pollution.
	5. I feel obliged to do my part to ensure my city has excellent air quality.
	6. I would feel guilty if I did not lodge a complaint about an air pollution incident to the environmental authorities.
Social norm (α = 0.799)	My colleagues promote a low-carbon lifestyle.
	2. People around me believe that a low-carbon lifestyle can reduce air pollution.
	3. My family supports me complaining about bad behaviour that pollutes the air (such as burning garbage, etc.).
	4. My family thinks I should use energy sparingly (e.g., save electricity, etc.) to slow down air pollution.
	5. My friends encourage me to volunteer for environmental activities.
	6. My friends like it when I talk to them about air pollution.
Self-efficacy (α = 0.617)	With my knowledge of the air pollution problem, I think I can persuade people around me to use low-carbon transport to reduce air pollution.
	2. I can personally help to reduce air pollution by saving energy.
	3. I think tackling air pollution is a national-level problem, while individuals can do nothing to tackle climate change.
	4. I personally feel that I can make a difference with regard to air pollution by engaging in emission-reduction behaviours.
	5. I feel like complaining about air pollution incidents to the authorities is beyond my capabilities.
	6. The use of the Internet to expose air pollution incidents is effective in combating air pollution.
	7. I feel I can help reduce air pollution by donating to environmental projects.

α = Cronbach’s alpha. Cronbach’s alpha is a measure of scale reliability where scores higher than 0.7 are considered reliable.

**Table A5:** The demographic and socioeconomic part of questionnaire A.

Demographic and Socioeconomic Conditions	
Question	Options
Permanent residence	(Province) _____ (City)_____
Gender	□Male □Female
Age	______
Student	□Yes □No
Education	□Uneducated □Primary education □Secondary education □Higher education
Whether you own a private car	□Yes □No
Occupation	□Students □Civil servant □Staff of public institutions □Self-employed entrepreneurs
	□Farmer □Company employee □Homemaker □Retirement □Unemployed □Worker
	□Other
Environmental protection worker ^a^	□Yes □No
Family monthly income (CNY)	□≤500 □501~2000 □2001~5000 □5001~10,000 □≥10,001

^a^ Environmental protection worker refers to the workers related to environmental protection, including students, teachers, environmental managers in the environmental professional field working indoors, as well as environmental cleaners who are mainly engaged in outdoor work.

**Table A6:** Questionnaire B.

Questionnaire B	
Child-rearing scale	
Although there are several qualities that people feel that children should have, every person thinks that some are more important than others. I am going to read you pairs of desirable qualities. Please tell me which one you think is more important for a child to have. The pairs of attributes are independence versus respect for elders, obedience versus self-reliance, curiosity versus good manners, and being considerate versus being well-behaved. Those who value “respect for elders,” “obedience,” “good manners,” and “being well behaved” score at the maximum of the scale. Those who value “independence,” “self-reliance,” “curiosity,” and “being considerate” score at the minimum.	
A independence	B respect for elders
A obedience	B self-reliance
A curiosity	B good manners
A being considerate	B being well behaved
